# Design of a randomized cross-over study evaluating effects of carbohydrate intake on glycemic control in persons with type 1 diabetes

**DOI:** 10.3389/fnut.2023.1114317

**Published:** 2023-03-13

**Authors:** Sofia Sterner Isaksson, Arndís F. Ólafsdóttir, Marcus Lind

**Affiliations:** ^1^Department of Molecular and Clinical Medicine, Sahlgrenska Academy, University of Gothenburg, Gothenburg, Sweden; ^2^Department of Medicine, NU Hospital Group, Uddevalla, Sweden; ^3^Department of Medicine, Sahlgrenska University Hospital, Gothenburg, Sweden

**Keywords:** type 1 diabetes mellitus, glycemic control, continuous glucose monitoring, medical nutrition therapy, carbohydrates (CHO)

## Abstract

**Introduction:**

Diet is an important factor in managing glycemic control in type 1 diabetes (T1D). Reducing carbohydrate intake may be important for stabilizing blood glucose levels in certain groups of patients with T1D. There are few studies examining the effects of a low carbohydrate diet in patients with T1D. The aim of this study is to investigate the effects of carbohydrate intake on glucose control in adults with T1D.

**Materials and methods:**

Adults with T1D (*N* = 54) and inadequate glycemic control (HbA1c ≥ 7.5%; 58 mmol/mol) were randomized in a cross-over design to a moderate carbohydrate diet (30 percent of total energy from carbohydrates) versus a traditional diabetes diet (50 percent of total energy from carbohydrates) for 4 weeks with a between wash-out period of 4 weeks. Masked continuous glucose monitoring was used throughout the study to evaluate effects on mean blood glucose levels, time-in-range, hypoglycemia, hyperglycemia, and glycemic variability. Diabetes treatment satisfaction, hypoglycemic confidence, and physical activity were measured using questionnaires during different phases of the trial. HbA1c, blood lipids, blood pressure, and ketone levels were also measured. The primary endpoint is the difference in mean blood glucose level between the diet periods. Study completion is anticipated during winter 2022.

**Discussion:**

The study seeks to increase knowledge about the effects of dietary carbohydrate intake on glycemic control and other health parameters in patients with T1D. If beneficial effects on mean blood glucose level without elevated risk of hypoglycemia or ketoacidosis are shown, a moderate carbohydrate diet may be a treatment option for people with T1D that have unsatisfactory blood glucose levels.

**Clinical Trials Registration:** www.clinicaltrials.gov, ID: NCT03400618.

## 1. Introduction

Good glycemic control in patients with type 1 diabetes (T1D) is related to decreased risk of complications and lower mortality (1–3). Mortality remains considerably higher in patients with T1D which could be decreased significantly if more patients reached target blood glucose levels (3, 4).

Diet is essential to achieve good glycemic control (5, 6). Mediterranean diet, Dietary Approaches to Stop Hypertension (DASH), and vegetarian dietary patterns are recommended by American Diabetes Association clinical practice guidelines for people with diabetes (5). Certain foods and food groups have been shown to have protective effects on cardiovascular risk (7–13). Carbohydrate counting is a method widely used in clinical practice, although the effectiveness of this method on glycemic control has been questioned (14–17).

Reducing dietary carbohydrates may be of importance for stabilizing glucose levels in certain groups of persons with T1D. If the insulin dose is incorrect by 20%, a greater effect on blood glucose will likely occur with greater carbohydrate content in the meal. Also, even during controlled conditions it is hard to predict an exact insulin dose making it difficult for patients to calculate the exact insulin dose to the amount of carbohydrates. This is because of the intraindividual variation in the uptake of insulin in the body of all available insulin sorts (18, 19). There are also many other factors that have an effect, such as physical activity during the past 24 h (20) making insulin doses hard to standardize.

Few studies have examined the effects of lower carbohydrate diets in patients with T1D and results are conflicting. A systematic review (21) examined the effects of LCDs (<45% of total energy) on glycemic control in eight studies of adults and children with type 1 diabetes. The studies were heterogenic and of small size and the results conflicting. In a large European observational study, they could see a trend that a lower intake of total carbohydrates was associated with lower HbA1c (22). This was also reported in another observational study in Denmark where higher intake of carbohydrates was associated with higher HbA1c in persons with T1D (23). A Finnish observational study reported that adherence to LCD was associated with lower BMI, lower variability of blood glucose measurements and lower diastolic blood pressure (24). A small study (*n* = 11) showed that a ketogenic diet (carbohydrate <55 g/day) was associated with excellent HbA1c levels [35 ± 4 mmol/mol (5.3 ± 0.4%)] and little glycemic variability, although it was also associated with dyslipidemia and a high number of hypoglycemic episodes (25). The studies that have shown beneficial effects on glycemic control with LCD are small (26–29) and/or not randomized and lacking control groups (29, 30).

The aim of this study is to analyze the effects of a moderate carbohydrate diet (30 percent of total energy) compared with a traditional diabetes diet (50 percent of total energy) on glycemic control and risk of ketoacidosis in patients with T1D.

## 2. Methods and analysis

### 2.1. Design and randomization

This is a randomized, open-label, cross-over clinical trial being conducted at four Swedish diabetes specialty centers. Enrolment commenced in March 2018 and study completion is expected during winter 2022. Patients were randomized 1:1 to either moderate carbohydrate or traditional diabetes diet for 4 weeks with an intermittent wash-out period of 4 weeks followed by the other intervention diet for 4 weeks. Expected study duration for each participant was 14–16 weeks, including a run-in period of 2–4 weeks. Eligible participants were identified from each diabetes center registry, and patients were invited to participate by written correspondence or telephone. Randomization was performed on site using a centralized web system with random permuted blocks of varying sizes. Each patient was assigned a unique coded subject ID at randomization.

### 2.2. Study participants

**Figure 1:**
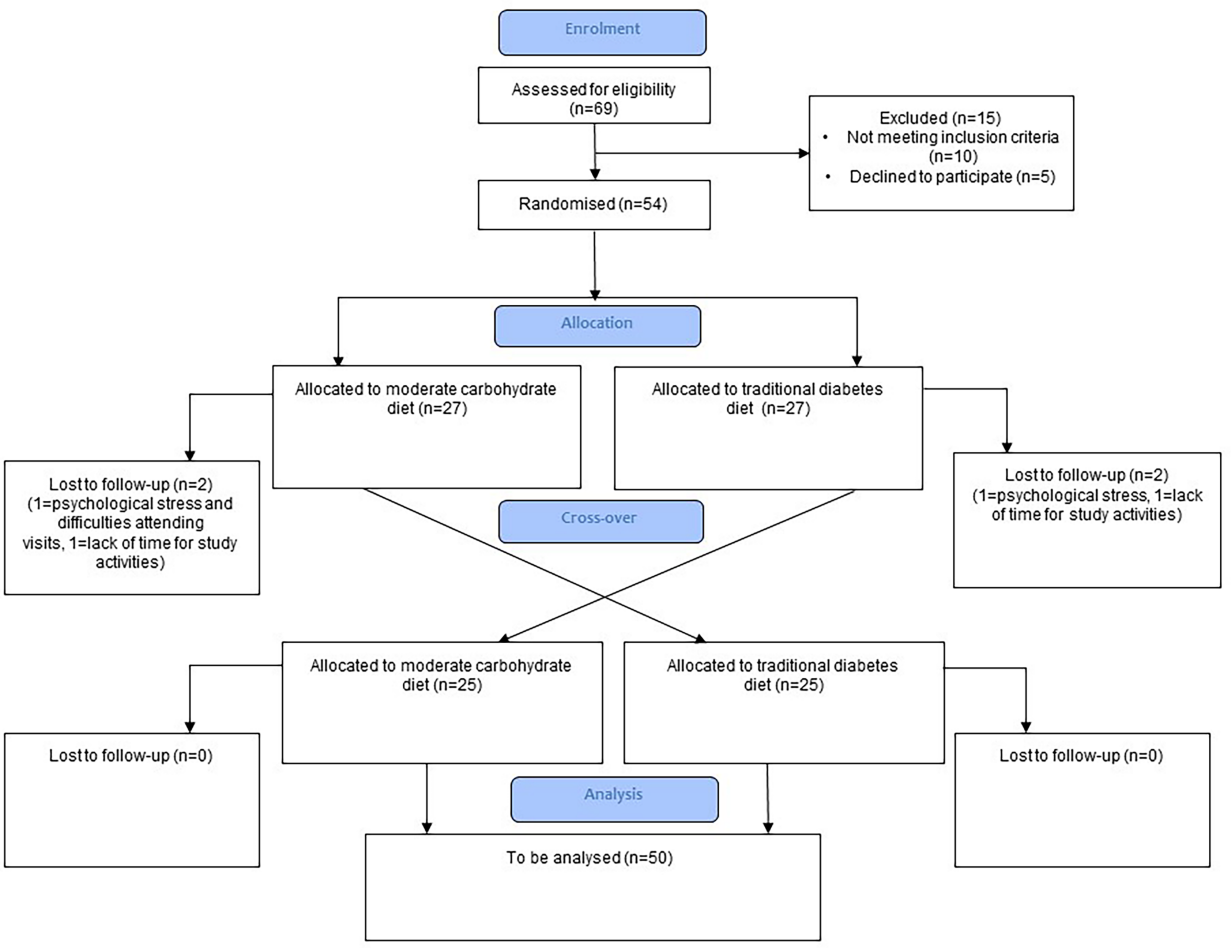
Flow diagram of participants during the cross-over study according to CONSORT.

Adults with T1D and HbA1c levels ≥58 mmol/mol (7.5% DCCT standard) were included in the study. Other inclusion and exclusion criteria are given in Table 1. A total of 69 subjects were screened and 54 randomized and included in the study. A flow diagram of participant recruitment during the cross-over study according to CONSORT is shown in Figure 1.

**Table 1:** Inclusion and exclusion criteria for participation in the study.

Inclusion criteria
1. Type 1 diabetes
2. Adults 18 years or older
3. Written informed consent
4. HbA1c ≥ 58 mmol/mol (7.5% DCCT standard)
**Exclusion criteria**
1. Pregnancy or planned pregnancy during the study period
2. Severe cognitive dysfunction or other disease as determined by the physician
3. Inability to comply with study diet (excluding foods common to each diet such as wholegrains, beans, lentils, fruit, and vegetables because of personal preferences)
4. Other disease
5. Diabetes duration <1 year
6. Planned change in diabetes treatment (e.g., commencing insulin pump or CGM) during the study period

The study was approved by the regional ethics committee of Gothenburg, Sweden (No. 473–17). All subjects received written and verbal information about the study and gave their written informed consent before inclusion.

### 2.3. Dietary interventions

Dietary interventions consisted of moderate carbohydrate diet: (30 percent of total energy from carbohydrates) and traditional diabetes diet with low glycemic index (50 percent of total energy from carbohydrates). Macronutrients in each diet are presented in Table 2.

**Table 2:** Macronutrients in the two intervention diets.

	Moderate carbohydrate diet	Traditional diabetes diet with low glycemic index
Carbohydrates (% of total energy)	30–40	50–60
Fat (% of total energy)	40–50	25–35
Protein (% of total energy)	20	15–20

Moderate carbohydrate diet included meat, fish, shellfish, eggs, vegetables, root vegetables, vegetable proteins, olive−/rapeseed oil, nuts and seeds, avocado, and margarine. Includes less sugar, bread, cereals, potatoes, rice, and pasta. Carbohydrates were mainly wholegrains and carbohydrates with low glycemic index.

Traditional diabetes diet included lean meat, fish, shellfish, vegetable proteins, beans, lentils, pasta, potatoes, rice, and grains. The recommended bread was wholegrain and with low glycemic index. Generally, it included a lot of vegetables and root vegetables as well as fruit and berries with low glycemic index (e.g., apple, orange, and pears), olive−/rapeseed oil, nuts and seeds, avocado, margarine, etc. This diet is in line with general nutrition recommendations.

Both diets were individualized by a dietitian to fit each subject in terms of energy (kcal) and macronutrient (carbohydrate, fat, and protein) intake and in line with current public nutrition guidelines. At clinical visits and each telephone contact, patients were encouraged to maintain diet in accordance with the protocol.

All diet material used in the study were made by a dietitian and included several recipes, different meal examples with carbohydrate content, whole day menus, and ideas for suitable foods, snacks, and meals suitable for each diet intervention. The same material was used at all 4 study sites but were individualized by the local dietitian depending on individual energy demands and personal preferences of participants.

All subjects recorded their food intake in a 4-days food diary at 3 times during the study. First during the run-in period to provide data for the dietitian to make the calculations and look at food preferences to be able to make the individual diet plans. To be able to measure adherence to the intervention diets the subjects were also instructed to record food intake for 4 days during the last 2 weeks of each intervention period. All food records will be calculated regarding macronutrient and energy intake by a dietitian. Protocol compliance and adherence were checked at each contact, and diet, blood glucose levels, and insulin dosages at each clinical visit.

### 2.4. Continuous glucose monitoring

All patients used a masked continuous glucose monitoring (CGM) system (Freestyle Libre Pro, Abbott Diabetes Care) during a run-in period of 2–4 weeks and then continuously for the rest of the study period for a total of 14–16 weeks. Data were collected from the CGM system at all clinical visits. Patients using CGM or Intermittent scanned Continuous Glucose Monitoring (isCGM) in their usual diabetes care continued to use this as well. Missing data from the masked CGM sensor could be replaced by data from patients regular, non-blinded CGM/isCGM device in the statistical analyses if available. All deviations such as which devices were used were carefully documented.

### 2.5. Insulin adjustments

Insulin adjustments were reviewed by a diabetes nurse or physician at the beginning of each treatment phase to ensure that participants adjusted insulin doses properly when changing diet. Clinical support was only provided at the start of each treatment phase, whereas during the evaluation period (last 14 days of each diet period) patients fully adjusted their doses on their own. Basal insulin and basal insulin dose in the insulin pump were adjusted by an algorithm primarily based on fasting blood glucose levels and overnight glucose profiles. At high fasting blood glucose levels and no tendency for nocturnal hypoglycemia the basal dose was increased in a stepwise fashion. Doses were reduced at low fasting blood glucose levels or nocturnal hypoglycemia. Glucose excursions at bedtime were also taken into consideration. For detailed algorithms see Supplementary material 1.

Patients were instructed to continue with the same strategy for dosing mealtime insulin as used in clinical practice (e.g., carbohydrate counting was continued if used). A dietitian educated patients about types of foods containing carbohydrates and provided similar support for both types of diets. A diabetes nurse or physician provided feedback at planned telephone contacts from glucose curves if apparent that mealtime doses were under- or overdosed to be taken into consideration for future mealtime insulin dosing. Patients were instructed to strive for 2-h post-meal glucose levels <10 mmol/l and pre-meal levels <7 mmol/l. Repeated glucose levels over these levels indicated that higher mealtime insulin doses were needed for that specific meal.

### 2.6. Measuring blood glucose level

Patients were advised to check glucose level (with CGM or self-monitoring of blood glucose, SMBG, depending on what the patient used) at least 4 times per day before meals and at bedtime according to diabetes guidelines and CGM system manufacturer recommendations. Patients were advised to test or check glucose levels more often if indicated. Measurement frequency from isCGM/CGM/SMBG devices were downloaded if available, otherwise patients were asked to self-report daily measurement frequency after each diet intervention period.

### 2.7. Clinical measurements

Height was measured at baseline. HbA1c was measured at baseline and before and after each diet intervention. Blood lipids (total, LDL and HDL cholesterol, apolipoproteins, and triglycerides), blood pressure, and weight were measured before and after each treatment period. Blood ketones were measured and recorded in a diary twice a week during the two diet periods by participants at home using a ketone measurement device.

### 2.8. Assessment of safety

Adverse events and severe adverse events were recorded at all visits and telephone contacts. The number of severe hypoglycemic events defined as unconsciousness due to hypoglycemia or requiring assistance were recorded. Time in low glucose values (<3.0 and <3.9) was recorded by CGM. Ketones, insulin doses, and occurrence of ketoacidosis were also recorded.

### 2.9. Questionnaires

The Diabetes Treatment Satisfaction Questionnaire (DTSQ) has been used in many diabetes therapy clinical trials and is a validated questionnaire consisting of 8 questions that measures aspects of satisfaction scored on a 7-point Likert scale, including domains of current treatment satisfaction, convenience, understanding, recommendations, and continuation of treatment (31, 32). Two separate items measure perception of hyperglycemia and hypoglycemia. Two versions were used: the DTSQs and DTSQc, where the DTSQs was used for recording current treatment satisfaction and the DTSQc for patients to retrospectively compare the two treatments. Patients completed the DTSQs before and after each diet period and the DTSQc after the last diet period.

The Hypoglycemia Confidence Scale is a validated, 9-item scale that evaluates patient confidence regarding their ability to prevent and address hypoglycemic events (33). Patients completed the questionnaire before and after each diet period at the study site. Study personnel aided with completing questionnaires if needed without influencing patient responses. Physical activity was measured at baseline and before and after each diet intervention by questionnaires and scales to calculate energy requirements for each diet. Questions included whether physical activity changed during the last 14 days and on a scale measuring physical activity from 1 to 10 as well as 24-h physical activity.

### 2.10. Trial procedures

**Figure 2:**
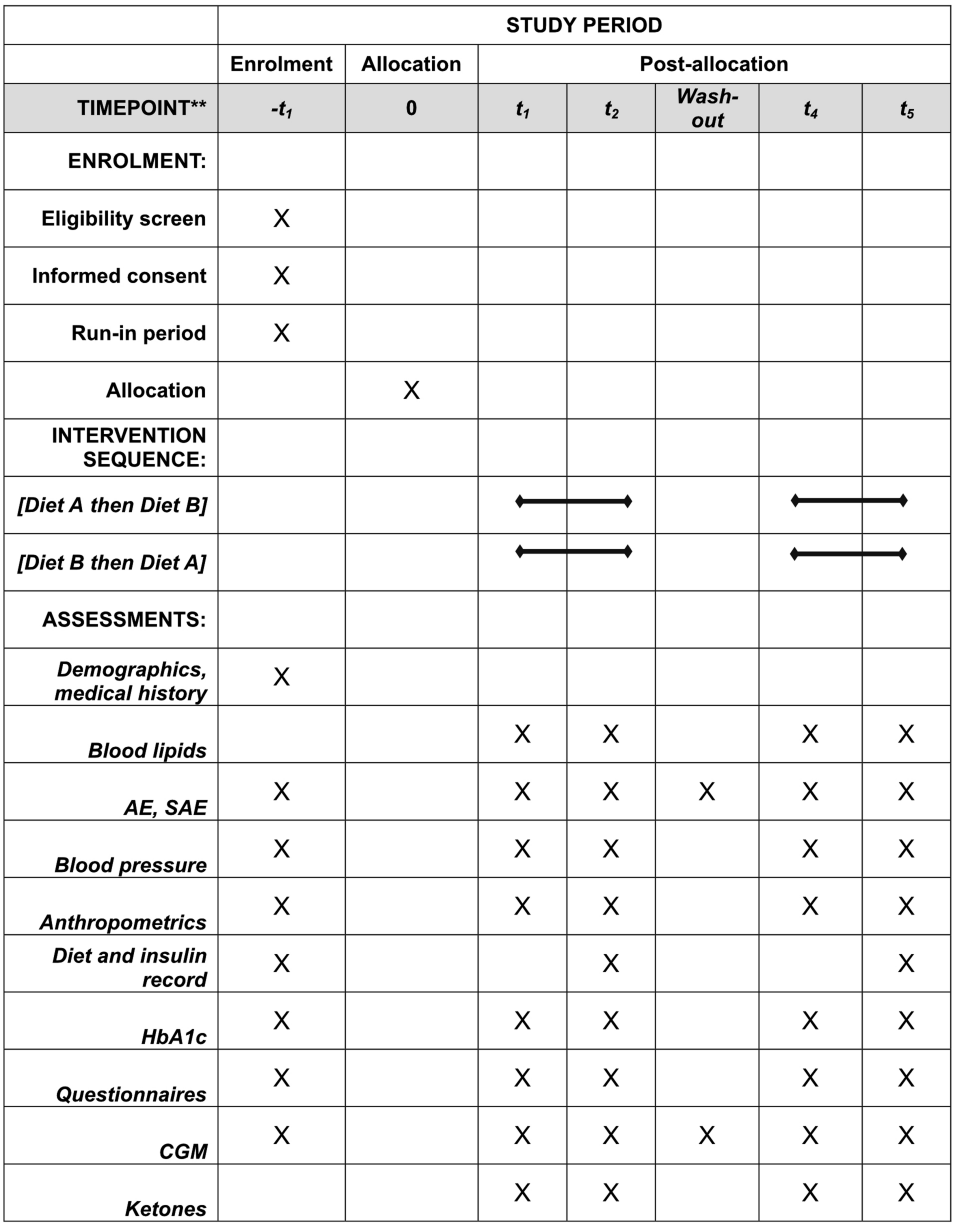
Enrolment schedule, interventions, and measurements according to Standard Protocol Items: Recommendations for Intervention Trials (SPIRIT) requirements. AE, adverse event; CGM, continuous glucose monitoring; SAE, severe adverse event.

Patients completed a total of 6 visits to the diabetes center. Visit 1 included information about the study and informed consent. Visit 2 was the start of a 2–4-week run-in period and included physical examination, start of masked CGM, instruction about recording food intake for 4 days, diet interview, and HbA1c measurement. Visit 3 included randomization and instructions from the dietitian and diabetes nurse for starting diet intervention. Patients meeting inclusion criteria, performing 2-week masked CGM, and completing food recording were randomized. Fasting blood samples, weight, and blood pressure were measured and questionnaires completed. Telephone follow-up was performed during the diet intervention at 1-, 4-, 7-, and 14-days post-intervention. At visit 4, the first intervention was completed and CGM data, fasting blood samples, weight, and blood pressure measured and questionnaires answered. At visit 5 the second intervention began with the same procedures followed as during the first intervention. At visit 6 the second intervention was completed, and data collected as with visit 4. Enrolment schedule, interventions, and measurements according to Standard Protocol Items: Recommendations for Intervention Trials (SPIRIT) requirements are shown in Figure 2. A checklist according to SPIRIT 2013 is also provided. See Supplementary material 2. All measurements and questionnaires were recorded in an eCRF-system (Medicase AB). The study was monitored by the Department of Molecular and Clinical Medicine, Institution of Medicine, Sahlgrenska Academy, University of Gothenburg.

### 2.11. Evaluation period and endpoints

Evaluations were performed during the last 2 weeks of each treatment phase when patients did not receive any clinical support regarding diet or insulin adjustments. The primary endpoint is the mean blood glucose level measured by CGM during the evaluation period. Secondary endpoints are the differences between the two diet interventions in standard deviations of blood glucose levels, time above range, time in range, weight, total, LDL, and HDL cholesterol, triglycerides, total insulin dose, DTSQs, DTSQc, and Hypoglycemia Confidence Score. All endpoints are shown in Table 3.

**Table 3:** Endpoints in the study.

Primary endpoint
Difference in mean blood glucose level between the two diet periods^1^
**Secondary endpoints**
The difference in standard deviation of blood glucose levels between the two diet periods^1^
The difference in time above range between the two diet periods (above 10.0 mmol/l and above 13.9 mmol/l respectively) ^1^
The difference in time in range (5.5–10.0 mmol/l and 3.9–10.0 mmol/l respectively) between the two diet periods^1^
The difference in weight between the two diet periods
The difference in total cholesterol between the two diet periods
The difference in LDL cholesterol between the two diet periods
The difference in HDL cholesterol between the two diet periods
The difference in triglycerides between the two diet periods
The difference in total insulin dose between the two diet periods
The difference in DTSQc score at the end of the study between the two diet periods
The difference in DTSQs scores between the two diet periods
The difference in hypoglycemia confidence scores between the two diet periods
**Other exploratory endpoints**
The difference in MAGE between the two diet periods^1^
The difference in HbA1c between the two diet periods
The difference in apolipoproteins between the two diet periods
**Safety endpoints**
The difference in the proportion of time with low blood glucose levels between the two diet periods (below 3.0 mmol/l and below 3.9 mmol/l respectively)^1^
The difference in the mean number of severe hypoglycemic events between the two diet periods defined as unconsciousness due to hypoglycemia or requiring assistance from another person
The difference in ketone levels between the two diet periods
Occurrence of ketoacidosis during the study period and differences between the two diet periods

^1^Measured with continuous glucose monitoring during the last 2 weeks of interventions.

### 2.12. Statistical methods

Appropriate statistical methods for cross-over trials will be applied. Normally distributed variables will be analyzed using linear mixed effects models with treatment, period and sequence as fixed effects, and subject as random effect. Log-normally distributed variables will be analyzed by similar means after transformation to log-scale. Treatment effects on log-scale will be exponentiated to obtain estimates of the fold-change between treatments. Other non-normally distributed continuous variables will be analyzed using linear mixed effects models without transformations, using robust standard errors to account for violations against distributional assumptions. Questionnaire scales will be treated as continuous in this regard. Binary and count variables will be analyzed using generalized linear mixed effects models with the same fixed and random effects as above, Poisson distribution and log-link. The corresponding treatment effect will be given as fold-change or relative risk between groups, as appropriate. Robust standard errors will be used to account for violations against distributional assumptions. Carry-over effects will be evaluated through the significance of treatment with period interactions, and by investigation of baseline, run-in, and wash-out values.

All efficacy analyses will be performed on the Full Analysis Set (FAS), defined as all randomized subjects who have registered CGM data for at least one of the two study periods. Primary and secondary efficacy analyses will also be performed on the Per-Protocol population (PP-population), consisting of all subjects in the FAS-population who have registered CGM data for both study periods, have completed the diet records of both study periods, have complied with the diets, and have no other protocol deviation that may have had any significant effect on the primary endpoint. The PP-population will be defined at a Clean File meeting before the database is locked. Safety analyses will be performed on the safety population, which consists of all randomized subjects who received the moderate carbohydrate diet. In safety analyses, patients will be classified by actual treatment taken, not to the randomized treatment.

The primary efficacy analysis will be the analysis of the mean difference in mean glucose levels measured by CGM during the last 2 weeks of the treatment period between moderate carbohydrate diet and traditional diabetes diet on the Full Analysis Set. The treatment effect will be estimated using linear mixed effects models for normally distributed variables as described above.

All statistical tests will be two-sided and conducted at the 5% significance level. To account for multiple testing, a sequential testing procedure will be employed. If a test gives a significant result at the 0.05 significance level, the total probability mass of 0.05 will be transferred to the next endpoint in the test sequence until a nonsignificant result is achieved. All these significant tests will be considered confirmatory. *p*-values and confidence intervals of remaining endpoints will be presented descriptively.

Statistical analyses will be performed using SAS statistical software version 9.4. A Statistical Analysis Plan with a detailed description of all planned analyses will be completed and signed before the database is locked. Once all study data are collected and entered, the database will be reviewed for completeness, accuracy, and consistency. At a formal Clean File meeting the database will be declared locked, and the analysis can start.

The target sample size was set to 52 subjects to detect a clinically relevant difference in mean glucose levels of 1 mmol/l between treatments, assuming a within subjects’ standard deviation of 2.5 mmol/l, paired *T*-test, 80% power, significance level *α* = 0.05. The standard deviation was estimated on data from the GOLD cross-over trial (34).

## 3. Discussion

This is a description of the protocol for a randomized controlled cross-over study investigating the impact of a moderate carbohydrate diet compared to a traditional diabetes diet with higher carbohydrate content on glycemic control and other health parameters in T1D.

Diet with moderate carbohydrate content (30 percent of total energy from carbohydrates) is considered safe in healthy populations and for people with type 2 diabetes but is quite different compared to the traditional diabetes diet (50 percent of total energy from carbohydrates) as generally recommended regarding amount of fat and carbohydrates (5, 6). A diet high in saturated fat and low in fiber, fruits and complex carbohydrates is not considered healthy (5, 6). Therefore, it was important to make both intervention diets healthy in terms of other nutrients not only macronutrients, which is why both diets included vegetables, root vegetables, nuts and seeds, fish and shellfish, legumes and unsaturated fats like olive or rapeseed oil as well as a focus on carbohydrate quality in both diets such as choosing wholegrains and foods rich in fiber with low glycemic index (7–13). Trying to make both intervention diets healthy and safe we choose a moderate amount of carbohydrates instead of a low- or very low carbohydrate diet. This was also because the diet should be tolerable and easy to incorporate into everyday life as well as making it easier to comply with for longer time periods, not only for the study period. Further, although it may seem like the difference in carbohydrate content is relatively small, the intervention group will get 40% less carbohydrates (e.g., 600 kcal instead of 1,000 kcal for a person with a daily energy intake of 2,000 kcal) which we consider to be a clinically relevant difference. Moreover, considering that participants may not fully comply to the diets we will also make the per protocol analyses including only the subjects that fully complied to the protocol and the diets.

The randomized design reduces the risk of confounding factors associated with various diets that can be related to blood glucose control and often difficult to account for in observational studies. Further, by using a cross-over design all participants were given the opportunity to try both diets and serve as their own controls eliminating interpatient variability. Because insulin therapy is different between all subjects, we made a structured management plan regarding changes in insulin regimen during the study to keep it as comparable as possible between the two diet periods. Use of CGM to measure blood glucose levels on all subjects continuously throughout the study provides detailed data about fluctuations in glucose control during changes in diet. Dietary interventions and all study material were developed by a registered dietitian and considered healthy and safe according to available evidence. Limitations include possible carry-over effects due to the cross-over design making interpretation of results potentially more difficult. Blinding of study participants was not possible because knowledge of carbohydrate content was crucial for patients. Existing knowledge, or preference, of low carbohydrate diets may lead to bias in favor of moderate carbohydrate diet versus traditional diabetes diet. It is difficult to measure diet compliance accurately as it is based on participant experience and self-reporting. Over- or underreporting of diet is common when it comes to food records or dietary assessments (35, 36).

The study period of 12 weeks and active intervention periods of each 4 weeks was planned to be relatively short since we wanted participants to comply to changes in diets and advanced study related procedures including food diaries, ketone levels and masked CGM. The main goal of the trial is to evaluate if different carbohydrate content in diet influences glucose levels. Moreover, the use of masked CGM-data for key endpoints capturing differences in glucose patterns continuously gives an opportunity to capture the current glucose effects during each treatment period. Hence, the current study will show if a lower content of carbohydrates in diet *per se* leads to more stable glucose levels and if ketone levels are influenced. However, it is important to notice that if the intervention diet leads to improved glucose control it will be essential to evaluate whether such a diet can be kept over long time periods with sustained effects on glucose control.

In summary, this study seeks to increase knowledge about the effects of dietary carbohydrate intake on glycemic control and other health parameters in patients with T1D. If beneficial effects on mean blood glucose level without elevated risk of hypoglycemia or ketoacidosis are shown, a moderate carbohydrate diet may be a treatment option for people with T1D that have unsatisfactory blood glucose levels. If shown to have no beneficial effects, this study will hopefully increase knowledge among healthcare professionals whether moderately decreasing carbohydrate intake is safe when educating patients about diet.

## Ethics statement

The studies involving human participants were reviewed and approved by the regional ethics committee of Gothenburg, Sweden (No. 473-17). The patients/participants provided their written informed consent to participate in this study.

## Author contributions

SS, AÓ, and ML designed the study. ML was the PI of the study. SS drafted the manuscript. All authors have reviewed and approved the final manuscript.

## Funding

This study is supported by The Healthcare Board, Region Västra Götaland, The Dr. P Håkansson Foundation and the Swedish state under the agreement between the Swedish government and the country councils, the ALF-agreement [ALFGBG-966173]. The sponsors did not have any role in the design and conduct of the study, collection, management, analysis and interpretation of the data and preparation, review, or approval of the manuscript.

## Conflict of interest

AÓ is a consultant for Nordic Infucare, and ML has received research grants from Eli Lilly, Novonordisk and been a consultant or received honoraria from Astra Zeneca, Eli Lilly, and Novonordisk, all outside the current study.

The remaining author declares that the research was conducted in the absence of any commercial or financial relationships that could be construed as a potential conflict of interest.

## Publisher’s note

All claims expressed in this article are solely those of the authors and do not necessarily represent those of their affiliated organizations, or those of the publisher, the editors and the reviewers. Any product that may be evaluated in this article, or claim that may be made by its manufacturer, is not guaranteed or endorsed by the publisher.

## Abbreviations

AE: adverse event
BMI: body mass index
CGM: continuous glucose monitoring
CONSORT: consolidated standards of reporting trials
DTSQ: diabetes treatment satisfaction questionnaire
HbA1c: glycated hemoglobin
isCGM: intermittent scanned continuous glucose monitoring.
SAE: serious adverse event
SD: standard deviation
SPIRIT: standard protocol items: recommendations for intervention trials.
T1D: type 1 diabetes mellitus.
