# Supplementary material for: Design of a randomized cross-over study evaluating effects of carbohydrate intake on glycemic control in persons with type 1 diabetes
Source: Front Nutr. 2023 Mar 13;10:1114317. doi: 10.3389/fnut.2023.1114317 (PMC10041710; doi:10.3389/fnut.2023.1114317)
Supplement: Supplementary file 1 [file Data_Sheet_1.PDF]

Supplementary Material

Design of a randomized cross-over study evaluating effects of carbohydrate intake on glycemic control in persons with type 1 diabetes

Sofia Sterner Isaksson*, Arndís F. Ólafsdóttir, Marcus Lind

*** Correspondence:** Sofia Sterner Isaksson: sofia.isaksson.2@gu.se

Basal insulin adjustments during the study

Insulin adjustments was performed by the patient him/herself according to the algorithm below. However, the physician or diabetes nurse supported the patient to follow these guidelines at planned telephone contacts according to protocol taking place at the start of each treatment phase. The patients received a form to record fasting glucose levels, nocturnal hypoglycaemia and insulin adjustments made of basal insulin doses.

Multiple Daily Injection (MDI)Therapy
The basal insulin dose was not recommended to be changed when initiating a diet. In each diet phase the basal insulin dose was evaluated from fasting glucose levels. During the first 2 weeks insulin glargine, insulin detemir and NPH insulins were evaluated every 3rd day. If the mean fasting glucose level was above 6.5 mmol/l and the patient did not have nocturnal hypoglycaemia or any fasting glucose level below 4.0 mmol/l the basal insulin dose shall be increased by 2 units in the FPG interval 6.5-10 mmol/l and 4 units if the mean level has been above 10 mmol/l. If the FPG level repeatedly has been 3-4 mmol/l or nocturnal hypoglycaemia has appeared or at any time FPG <3.0 mmol/l the basal insulin dose shall be reduced by 2-4 units. For insulin degludec the same algorithm shall be used every 5th day. If the patient or caregiver wants to reduce an insulin dose even earlier than every 3rd day due to safety reasons such as fear of hypoglycaemia this shall be made, recommended by 2-4 units. If the patient repeatedly had clear postprandial hyperglycaemia after the evening meal and high glucose levels at bedtime and the evening meal prandial dose is decided to be increased, this can be another reason for awaiting increasing the basal insulin dose although FPG-levels are increased. If the patient has daytime hypoglycaemias that are clearly judged to be due to too high basal insulin dose that cannot be compensated by e.g., suitable meals an individual judgement must be made by the patient/caregiver if the basal insulin dose shall be reduced/not increased although FPG may be increased. In summary the above basal insulin algorithm is a recommendation to follow, but safety must always be prioritised and if the patient/care-giver judges that the algorithm should not be followed from a safety perspective in certain instances this should be done.

Insulin Pump Therapy
For persons on insulin pumps a similar algorithm were used every 3rd day. The basal insulin dose overnight shall be increased by an average by 0.1 units/hour if FPG 6.5-10 mmol/l and by an average of 0.2 units/hour if FPG >10 mmol/l. In correspondence the basal overnight insulin dose shall be reduced by 0.1-0.2 units/hour at nocturnal hypoglycaemia or low FPG-levels (see description above

for basal insulins). Also, in correspondence the patient and caregiver always have the right from a safety perspective to reduce or skip enhancing the basal insulin dose if fear of hypoglycaemia. For patients on insulin pumps individual judgement will be made if the basal insulin dose daytime shall be adjusted. During weeks 2-4 in each treatment phase the above insulin adjustments will be made every 5th day by the patient him/herself. The same general recommendations for deviating from this algorithm for insulin pump users shall be applied for individuals using MDI.
