# Supplementary material for: Design of a randomized cross-over study evaluating effects of carbohydrate intake on glycemic control in persons with type 1 diabetes
Source: Front Nutr. 2023 Mar 13;10:1114317. doi: 10.3389/fnut.2023.1114317 (PMC10041710; doi:10.3389/fnut.2023.1114317)
Supplement: Supplementary file 3 [file Data_Sheet_3.pdf]

Supplementary Material

Design of a randomized cross-over study evaluating effects of carbohydrate intake on glycemic control in persons with type 1 diabetes

Sofia Sterner Isaksson*, Arndís F. Ólafsdóttir, Marcus Lind

*** Correspondence:** Sofia Sterner Isaksson: sofia.isaksson.2@gu.se

Supplementary Material

Supplementary Material 1: Basal insulin adjustments during the study

Supplementary Material 2: SPIRIT 2013 checklist
